# Relationship Between Dietary Knowledge, Socioeconomic Status, and Stroke Among Adults Involved in the 2015 China Health and Nutrition Survey

**DOI:** 10.3389/fnut.2021.728641

**Published:** 2021-09-27

**Authors:** Ruojun Xiang, Xiaoya Zhai, Qiujin Zhang, Zhihua Wang

**Affiliations:** ^1^School of Public Health, Guilin Medical University, Guilin, China; ^2^Guangxi Key Laboratory of Tumor Immunology and Microenvironmental Regulation, Guilin Medical University, Guilin, China; ^3^Department of Immunology, School of Basic Medicine, Guilin Medical University, Guilin, China

**Keywords:** stroke, dietary knowledge, socioeconomic status, CHNS, public health

## Abstract

Stroke is a public health threat that requires urgent attention in China. Nutrients have individual significant impacts on the prevalence of stroke. However, little research has been conducted on the impact of dietary knowledge on stroke and whether the impact is potentially heterogeneous under the effect of socioeconomic status. This study used the 2015 Chinese Health and Nutrition Survey to explore the impact of dietary knowledge and socioeconomic factors on populations suffering from stroke. Results indicated that risk of stroke decreased significantly with increasing dietary knowledge score. Additionally, the impact of dietary knowledge scores on the prevalence of stroke has obvious heterogeneity. First, dietary knowledge scores significantly influenced low-income groups and individuals with low educational levels. Second, the risk of stroke in females is more affected by dietary knowledge. Third, for people living in different areas, dietary knowledge determines whether rural populations suffer from stroke.

## Introduction

Stroke refers to an acute cerebral blood circulation disorder, and cerebral ischemic and hemorrhagic damage are its main clinical manifestations. It has a high incidence rate and long course and leads to disability and mortality (1). To date, as many as 1.88 million Chinese residents die of stroke each year (2), which is four or five times the number in developed countries, such as Europe and the United States (3). Its death rate is much higher than the world average and has become the leading cause of death among Chinese populations (4). According to China's National Stroke Screening Data, the standardized incidence rate of first stroke increased from 0.40% in 1993 to 1.23% in 2013 and has continued to increase at an annual rate of ~8.7%. Moreover, the diseased population tends to become younger (5). Stroke mortality and morbidity have a high disease burden, costing China's economy up to 400 billion yuan each year, and this amount increases annually (6). The global stroke epidemic is a public health crisis that requires the urgent attention of all countries.

Studies have shown that excessive intake of saturated fat and cholesterol (7), high-sugar diet (8), and low level of total dietary fiber intake (9) are the main dietary factors increasing the risk of stroke. Previous studies mostly focused on the impact of a single foodstuff or nutrient on stroke (10), no prior studies considered the interactions among a population's dietary knowledge, overall dietary structure, and prevalence of stroke. Dietary structure can comprehensively represent nutrients, calories, and trace elements consumed by an individual (11). The influence of individuals' level of dietary knowledge is reflected by their food choices and preferences (12). The World Food Program pointed out that the measurement standard of high-quality diet is the frequency of daily consumption of various food products (13), and A single dietary structure can increase the prevalence of cardiovascular diseases in a population (14).

To address the impact of chronic diseases, such as stroke, the Chinese government proposed an appropriate dietary in the “Healthy China 2030.” This plan formulates and implements a national nutrition plan, publishes dietary guidelines suitable for different population characteristics, and aims to improve dietary literacy and promotes the formation of scientific eating habits and maintenance of a good lifestyle (15).

The main way to improve people's diet is to enhance people's knowledge of literacy diet (16). In the 1960s, the British scholar Kirster proposed the Knowledge, Attitude and Practice Theory Model (KAP), which is the most common model used in explaining the influence of personal knowledge and beliefs on healthy behaviors. The theory divides change in human behavior into three continuous processes: acquiring knowledge (Knowledge), generating belief (Attitude), and forming behavior (Practice). Through learning, a population acquires relevant health knowledge and skills and gradually form healthy beliefs and attitudes, which promote healthy behaviors. According to this theory, correct and rich dietary knowledge is essential to promoting healthy diet to a population for the reduction of stroke prevalence.

The dietary structures of a population are affected by many factors, such as socioeconomic status (SES), urban and rural settings, and educational level (17). Dietary patterns vary greatly among different socioeconomic groups (18), and unhealthy diet patterns are mostly observed in low-income groups (19). This situation may be related to the traditional Chinese food culture. People with low SES are inclined to choose affordable and high-calorie food because they barely receive scientific dietary guidance, but such food often lacks nutrients (20). This behavior further affects their health.

The detailed relationships among stroke prevalence, dietary knowledge, and SES are still unclear. Most relevant studies are led by the United States and other Western countries, and such studies are limited in China. In this study, we used the 2015 China Health and Nutrition Survey (CHNS) data to explore this issue. We conducted a survey to collect detailed information on the individual characteristics of populations, diet, smoking and drinking histories, illnesses, and injuries. These pieces of information are useful in comprehensively investigating the impact of dietary knowledge and SES factors on the risk of stroke. Overall, after controlling demographic characteristics, geographic characteristics, and health-related behaviors that affect stroke, we found that dietary knowledge and SES have significant impacts on residents suffering from stroke. When the score for dietary knowledge increases by one point, the probability of residents suffering from stroke decreases by 4%. The performance of stroke in individuals is characterized by the higher the income, the lower the risk of stroke, when SES is considered. People with jobs are less likely to have a stroke, and the level of education is positively correlated with the incidence of stroke.

The present study has two main contributions: First, stroke is the leading cause of years of life lost due to premature mortality in the Chinese population (2), and it has become a focus of academic research in recent years. High-sugar and high-fat food and some nutrients have significant impacts on stroke (7–9), but understanding of the impact of dietary knowledge on stroke remains limited. Second, evidence suggests that SES is among the most important factors affecting health, causing widespread societal concern. Research has been carried out on the impact of SES on chronic non-communicable diseases (17, 21, 22), but few studies on the effects of the above factors on stroke considered dietary knowledge and SES. Dietary knowledge literacy is a key element in determining individual healthy behaviors. Studying its impact on stroke can help individuals gain correct dietary knowledge independently. This work will generate novel insights as to how to reduce the risk of various diseases.

## Materials and Methods

### Data and Variables

#### Data

The data used in this study were obtained from the “China Health and Nutrition Survey” (CHNS) ^1^, which is a longitudinal follow-up survey conducted by the Chinese Center for Disease Control and Prevention and the University of North Carolina (23). The survey started in 1989 and adopted multistage stratified cluster random sampling in 1991, 1993, 1997, 2000, 2004, 2006, 2009, 2011, and 2015. The scope of the investigation was extended from nine provinces (Heilongjiang, Liaoning, Shandong, Jiangsu, Henan, Hunan, Hubei, Guangxi, and Guizhou) to 15 provinces and cities. In 2011, three municipalities directly under the Central Government, Beijing, Shanghai, and Chongqing were added. In 2015, three provinces, namely, Shaanxi, Yunnan, and Zhejiang, were added. The current study included 7,319 family households and 20,914 individuals. This survey includes personal demographic data, work, income, family conditions, dietary knowledge, education, health, and public health (23), fulfilling the requirements of the present study. By using the CHNS2015 individual questionnaire survey module, adults with ages of over 18 were selected, and 8,561 survey subjects were finally included in the study through information matching and cleaning of missing values.

#### Variables

The stroke prevalence data in this study were obtained using the CHNS2015 questionnaire. The answer to the question “Has a doctor ever given you the diagnosis of stroke or transient ischemic attack?” was used as the basis for determining whether a stroke has occurred. According to the respondents' answers, this study constructed two dummy variables that represent whether a respondent had stroke and obtained a binary variable “whether the individual was diagnosed with a stroke” as the dependent variable of this study.

The explanatory variable of interest represented diet-related issues. A total of 17 questions related to dietary knowledge were obtained from the CHNS2015, and a dietary knowledge score sheet was summarized (Appendix in Supplementary Table 1). Each question requires the respondent to select “strongly disagree,” “disagree,” “neutral,” “unknown,” “agree,” or “strongly agree” depending on their personal circumstances. According to the existing literature (20), we re-assigned the above options, and the generated value was between 0 and 2, indicating the correctness of the answer (“T/F” column in Appendix in Supplementary Table 1): a score of 0 represented “strongly disagree,” “disagree,” and “unknown”; [1] represented “neutral”: and [2] represented “Agree” and “strongly agree.” Finally, the total score of dietary knowledge was obtained by summarizing the scores for the 17 questions. Among them, the highest score for dietary knowledge is 34. A high score indicated abundant dietary knowledge. The validity and reliability of the dietary knowledge questionnaire have been evaluated (24). The internal reliability of the questionnaire was evaluated with a Cronbach's alpha test. The Cronbach's alpha of the questionnaire was 0.75, which was greater than the accepted standard of 0.7, indicating that the questionnaire had high internal reliability.

We used five questions about food preference (see Appendix in Supplementary Table 2) as the criteria for characterizing the dietary behaviors of respondents. Respondents were required to select “like very much,” “like,” “neutral,” and “dislike,” “dislike very much,” and “does not eat this food” according to their personal preferences. With reference to the existing literature, we re-assigned the above options, and the values generated were between 0 and 5, which indicated the correctness of the answers (“P/N” column in Appendix in Supplementary Table 1) (25). Finally, the sum of the scores of the five questions were obtained and used in obtaining the total score of dietary knowledge. The highest score was 25, and a high score in indicated that a respondent is likely to have scientific dietary behavior.

In this paper, Cronbach's alpha test was performed on the dietary preference questionnaire, and the reliability coefficient was 0.70, which was in line with the accepted standard of 0.70, indicating that the questionnaire had high internal reliability.

This study selected two questions in the survey to represent whether the respondents are aware of dietary guidelines (26): “Do you know about the Chinese Pagoda or the Dietary Guidelines for Chinese Residents?” and “Do you proactively look for nutrition knowledge?”. The “Chinese Pagoda” and “Dietary Guidelines for Chinese Residents” were published by the Chinese Nutrition Society, aiming to provide healthy diet guidelines to Chinese people aged over 2 years old, promote the health of the population, prevent non-communicable diseases, and help the population in maintain a healthy weight (27). Based on the respondents' answers to the above two questions, we constructed two dummy variables to indicate whether they are aware of the “Chinese Pagoda” and “Dietary Guidelines for Chinese Residents” and whether they will actively acquire dietary knowledge.

The socioeconomic status of each respondent might have an impact if the risk of stroke. According to Vlismas (17), we selected marital status, educational level, work status, and income in the CHNS2015 personal questionnaire as reference indicators for individual socioeconomic factors.

Control variables included demographic, geographic, and health-related behavioral variables. Demographic characteristics include gender and age; geographic characteristics variables include geographic location (eastern, central, and western regions) and place of residence (urban or rural); and health-related behavioral variables include smoking and drinking (21, 28–31) Appendix in Supplementary Table 3. details specific processing methods and explanations of these variables.

### Data and Variables Statistical Analysis

Given that this study aimed to explore the impacts of SES and dietary knowledge on stroke, we established a hypothesis that all socioeconomic, dietary knowledge, and potential control variables in the CHNS are related to stroke. To understand the impacts of the above variables on stroke, we constructed the following model according to the research of Luo and Waite (32) and House et al. (33):


$$Stroke_{i}\;=\;\alpha \;+\;\beta Diet_{i}\;+\;\gamma SES_{i}\;+\;\theta X_{i}\;+\;\varepsilon_{i}$$

Stroke_*i*_ indicated whether the *ith* resident had suffered a stroke, and Diet_*i*_ indicates the dietary knowledge and dietary preference scores of the *ith* resident. SES_*i*_ is a collection of socioeconomic variables, including marital status, educational level, work status, and income of the *ith* resident, and X*i* represents the set of control variables for the *ith* resident, including demographic variables (gender and age), geographical variables (geographic location-eastern, central, and western regions and place of residence-urban or rural), and health-related behavioral variables (whether smoking or drinking and whether the individual has a history of hypertension). The variable ε_*i*_ is the random error term in the regression.

In order to measure the relative influence of various factors on whether residents suffer from stroke, this study uses standardized regression coefficients. The standardized regression model is:


$${\hat{Stroke}}_{i}\;=\;{\hat{b}}_{1}Diet_{i}+{\hat{b}}_{2}SES_{i}\;+\;{\hat{b}}_{3}X_{i}\;+\;\varepsilon_{i}$$

Typically, ${\hat{b}}_{j}$ is called standardized coefficients or β coefficients. The meaning of this coefficient is that if the independent variable is increased by 1 times standard deviation, then ${\hat{\text{Stroke}}}_{i}$ will change by ${\hat{b}}_{j}$ times standard deviation.

## Results

Table 1 shows the basic descriptive statistics of the main variables used in this study. The number of strokes in the sample accounted for ~1.35%. The average score of dietary knowledge is 26.9, near the full score of 34, showing that the current population has a relatively high degree of dietary knowledge. Dietary preference has an average score of 8.9, which is much lower than 25. We speculate that although the respondents have good dietary knowledge, they are more inclined to prefer food that meet their own preferences over healthy food. Regarding the two questions about whether the respondents have knowledge about “Chinese Pagoda” or “Dietary Guidelines for Chinese Residents” and whether they would actively learn or collect knowledge related to dietary knowledge, only 29.3% of residents know relevant content, and 29.4% of the respondents would take the initiative to understand and collect diet-related knowledge. The sample has more women than men, and nearly 88% are married. According to age structure, the age distribution has a range of 18–94 years, with an average of ~50 years old. The average educational level of the sample is greater than the level of compulsory education, and 50% of the respondents are employed at the time of the questionnaire survey. The average family income per capita is 15,489.4 yuan. Smoking and drinking behaviors accounted for 30.3 and 28.9% of the samples, respectively. The prevalence of hypertension (34), which has been confirmed as a risk factor for stroke, accounted for 15.4% of the sample.

**Table 1:** Summary statistics.

**Variables**	***N***	**Mean**	**Std. Dev**.	**Min**	**Max**
Stroke	8,561	0.013	0.112	0	1
Diet knowledge	8,561	26.855	5.120	0	34
Food preference	8,561	8.904	2.509	0	19
Gender	8,561	0.477	0.499	0	1
Job	8,561	0.507	0.500	0	1
Alcohol	8,561	0.303	0.459	0	1
Smoke	8,561	0.289	0.453	0	1
Education	8,561	2.367	0.968	1	4
Age	8,561	50.747	14.465	18	94
Age^∧^2	8,561	2784.435	1481.052	324	8,836
Having adequate dietary knowledge literacy	8,561	0.294	0.456	0	1
Income	8,561	15489.443	22545.164	−106,875	439,000
Residence	8,561	0.611	0.488	0	1
Marriage	8,561	0.88	0.325	0	1
Knowing about CFP/DGGCR	8,561	0.293	0.455	0	1
Hypertension	8,561	0.154	0.361	0	1

*The unit of income is CNY, which means annual household income*.

### Effect of Dietary Knowledge Score on the Prevalence of Stroke

We investigated the impact of residents' dietary knowledge scores on the prevalence of stroke under the premise of controlling other factors. Table 2 shows the estimated results of the model. Models 1–4 reported the impacts of dietary knowledge scores on the prevalence of stroke in residents after the addition of related variables, such as dietary knowledge score and preference score; control variables related to socioeconomic factors; individual characteristic control variables; and disease-related risk factors. Table 2 shows that the influence coefficient of dietary knowledge score on stroke prevalence is negative, indicating that that the high the dietary knowledge scores of residents significantly reduce prevalence of stroke all when all other things are equal. We found that in every one-point increase in dietary knowledge, the probability of residents suffering from stroke decreases by 4%. The dietary preference score is negatively correlated with the probability of stroke. Actively understanding or collecting information related to dietary knowledge will significantly reduce the possibility of stroke, whereas only knowing “Chinese Pagoda” or “Dietary Guidelines for Chinese Residents” is positively correlated with stroke, but the difference is non-significant.

**Table 2:** OLS estimates-effect of diet score on stroke.

	**(1)**	**(2)**	**(3)**	**(4)**
Diet knowledge	−0.044^***^	−0.038^***^	−0.035^***^	−0.040^***^
	(0.00)	(0.00)	(0.00)	(0.00)
Food preference	−0.060^***^	−0.050^***^	−0.031^***^	−0.024^**^
	(0.00)	(0.00)	(0.00)	(0.00)
Knowing about CFP/DGGCR	0.011	0.014	0.013	0.016
	(0.00)	(0.00)	(0.00)	(0.00)
Having adequate dietary knowledge literacy	−0.025^*^	−0.028^**^	−0.027^**^	−0.032^**^
	(0.00)	(0.00)	(0.00)	(0.00)
Gender			−0.047^***^	−0.055^***^
			(0.00)	(0.00)
Age			−0.277^***^	−0.279^***^
			(0.00)	(0.00)
Age∧2			0.383^***^	0.338^***^
			(0.00)	(0.00)
Residence			0.005	0.006
			(0.00)	(0.00)
Marriage			0.013	0.018
			(0.00)	(0.00)
Job		−0.085^***^	−0.045^***^	−0.037^***^
		(0.00)	(0.00)	(0.00)
Education		0.015	0.031^**^	0.031^**^
		(0.00)	(0.00)	(0.00)
Income		−0.007	−0.014	−0.017
		(0.00)	(0.00)	(0.00)
Alcohol				−0.049^***^
				(0.00)
Smoke				0.026^*^
				(0.00)
Hypertension				0.146^***^
				(0.00)
Constant	0.063^***^	0.062^***^	0.082^***^	0.088^***^
	(0.0078)	(0.01)	(0.02)	(0.02)
Observation	8,561	8,561	8,561	8,561

*Robust standard errors are reported in parentheses*.

* *10% significance level*.

** *5% significance level*.

*** *1% significance level*.

Regarding the relationship between socioeconomic variables and stroke prevalence, the risk of stroke decreases with increasing income. From a work perspective, working people are less likely to have a stroke, which may be related to the majority of non-working people who are retired and elderly people (35). Educational level is positively correlated with stroke incidence. From the perspective of the relationship between individual characteristic variables and the prevalence of stroke, men are significantly more likely to suffer from the disease than women, and this finding may have a certain correlation with men's life and eating habits (36). From the perspective of age structure, a significant inverse U relationship between stroke incidence and age was observed. We also found that smokers are more likely to have a stroke, and patients with hypertension are significantly more likely to suffer from a stroke than non-hypertensives.

### Robustness Checks

To verify whether the estimation result is sensitive to the estimation method, we used the Logit estimation method to estimate Equation (2) (Table 3). Table 3 shows that the coefficient of dietary knowledge estimated by Logit on stroke is still significantly negative. Increase in dietary knowledge score reduces a resident's risk of stroke. The influence of socioeconomic factors on stroke is roughly equivalent to the coefficient estimation result obtained by the OLS method. Therefore, the results show that the cross-sectional data estimation results are basically robust.

**Table 3:** Robustness checks.

	**(1)**	**(2)**	**(3)**	**(4)**
Diet knowledge	−0.041^***^	−0.035^**^	−0.035^**^	−0.041^***^
	(0.01)	(0.01)	(0.01)	(0.02)
Food preference	−0.168^***^	−0.137^***^	−0.085^**^	−0.057
	(0.03)	(0.03)	(0.04)	(0.04)
Having adequate dietary knowledge literacy	0.196	0.309	0.330	0.445
	(0.27)	(0.28)	(0.28)	(0.29)
Knowing about CFP/DGGCR	−0.576^**^	−0.664^**^	−0.733^**^	−0.868^***^
	(0.29)	(0.29)	(0.30)	(0.31)
Gender			−1.002^***^	−1.071^***^
			(0.24)	(0.27)
Age			0.315^***^	0.199^**^
			(0.10)	(0.09)
Age^∧^2			−0.002^***^	−0.001
			(0.00)	(0.00)
Residence			0.026	0.098
			(0.22)	(0.23)
Marriage			0.069	0.145
			(0.33)	(0.34)
Job		−2.133^***^	−1.409^***^	−1.315^***^
		(0.32)	(0.34)	(0.35)
Education		0.101	0.202^*^	0.227^*^
		(0.11)	(0.11)	(0.12)
Income		−0.000	−0.000	−0.000^**^
		(0.00)	(0.00)	(0.00)
Alcohol				−0.741^***^
				(0.26)
Smoke				0.448^*^
				(0.24)
Hypertension				2.008^***^
				(0.23)
Constant	−1.795^***^	−1.830^***^	−13.982^***^	−10.813^***^
	(0.38)	(0.44)	(3.20)	(3.08)
Observation	8,561	8,561	8,561	8,561

*Robust standard errors are reported in parentheses*.

* *10% significance level*.

** *5% significance level*.

*** *1% significance level*.

### Heterogeneity Analysis of the Influence of Dietary Knowledge Score on the Prevalence of Stroke

Several reports have shown that the heterogeneity of related factors influencing stroke (37–39). To investigate whether dietary knowledge scores have heterogeneity in the impact of stroke prevalence, we studied the heterogeneity of dietary knowledge scores on stroke events in different income groups, different educational levels, different genders, and urban and rural residents. Table 4 shows the heterogeneous effects of dietary knowledge scores on the incidence of stroke in groups with different incomes and educational levels.

**Table 4:** OLS estimates-effect of diet score on stroke by income group & education group.

**Subsample**	**(1)**	**(2)**	**(3)**	**(4)**
	**Income group**		**Education group**	
	**Low-income**	**High-income**	**Low**	**High**
Diet knowledge	−0.064^***^	−0.006	−0.048^***^	−0.020
	(0.00)	(0.00)	(0.00)	(0.00)
Food preference	−0.023	−0.026	−0.025^*^	−0.016
	(0.00)	(0.00)	(0.00)	(0.00)
Gender	−0.065^***^	−0.045^**^	−0.064^***^	−0.047^**^
	(0.00)	(0.00)	(0.00)	(0.00)
Job	−0.036^**^	−0.040^**^	−0.035^**^	−0.041^**^
	(0.00)	(0.00)	(0.00)	(0.00)
Alcohol	−0.072^***^	−0.023	−0.073^***^	−0.012
	(0.00)	(0.00)	(0.00)	(0.00)
Smoke	0.034^*^	0.019	0.034^*^	0.010
	(0.00)	(0.00)	(0.00)	(0.00)
Education	−0.006	0.074^***^	0.006	0.043^**^
	(0.00)	(0.00)	(0.00)	(0.00)
Age	−0.223^**^	−0.354^***^	−0.188^**^	−0.461^***^
	(0.00)	(0.00)	(0.00)	(0.00)
Age^∧^2	0.269^***^	0.430^***^	0.236^**^	0.532^***^
	(0.00)	(0.00)	(0.00)	(0.00)
Having adequate dietary knowledge literacy	−0.015	−0.043^**^	−0.053^***^	−0.001
	(0.01)	(0.00)	(0.00)	(0.00)
Income	0.004	−0.012	−0.023^*^	−0.014
	(0.00)	(0.00)	(0.00)	(0.00)
Residence	0.016	−0.009	0.006	−0.002
	(0.00)	(0.00)	(0.00)	(0.00)
Marriage	0.017	0.022	0.005	0.040^**^
	(0.01)	(0.01)	(0.01)	(0.01)
Knowing about CFP/DGGCR	0.011	0.013	0.020	0.004
	(0.01)	(0.00)	(0.00)	(0.00)
Hypertension	0.164^***^	0.131^***^	0.138^***^	0.160^***^
	(0.01)	(0.00)	(0.00)	(0.01)
Constant	0.096^***^	0.050^**^	0.098^***^	0.062^**^
	(0.02)	(0.02)	(0.02)	(0.03)
Observation	4,281	4,280	5,030	3,531

*Robust standard errors are reported in parentheses*.

* *10% significance level*.

** *5% significance level*.

*** *1% significance level*.

First, on the basis of income level, the sample was divided into two groups: low-income and high-income groups, and estimated regression results were obtained. According to the study of Apouey et al. (40), income was ranked from low to high. Residents before the median were defined as “low-income groups,” whereas those behind were defined as “high-income groups.” We found an interesting finding from the regression results of income grouping in Table 4: the dietary knowledge score has a greater impact on low-income people suffering from stroke, with a coefficient of −0.064, but it has no significant impact on high-income people with stroke.

Second, we considered whether the impact of dietary knowledge scores on people suffering from stroke vary by educational level. Learning from Ning and Guangjie (41), we divided the sample into two groups according to educational level. People with the highest degree of high school were included in the low-education group, and those with university education or above were included in the high-education group. The impacts of dietary knowledge scores on stroke in different educated groups were investigated. The group regression based on education in Table 4 shows that the dietary knowledge score has an extremely significant positive impact on the low education group, with a coefficient of 0.048.

Whether the impacts of dietary knowledge scores on patients with stroke vary by gender was also investigated. Table 5 indicates that regardless of whether a man suffers from a stroke seems to be unaffected by dietary knowledge score, but increase in dietary knowledge score has a stronger impact on the reduction in stroke incidents in women. From the perspective of the differences in the impacts of dietary knowledge scores on stroke among urban and rural residents, the dietary knowledge scores have a significant impact on the reduction in stroke incidents among rural populations, with a coefficient of 0.056. That is, in cities and rural areas within the same prefecture-level city, if residents have roughly the same level of dietary knowledge, rural residents will have a lower risk of stroke.

**Table 5:** OLS estimates-effect of diet score on stroke by gender group & residence group.

**Subsample**	**(1)**	**(2)**	**(3)**	**(4)**
	**Gender group**		**Residence group**	
	**Male**	**Female**	**Urban**	**Rural**
Diet knowledge	−0.023	−0.077^***^	−0.010	−0.056^***^
	(0.00)	(0.00)	(0.00)	(0.00)
Food preference	−0.029^*^	−0.021	0.019	−0.052^***^
	(0.00)	(0.00)	(0.00)	(0.00)
Gender	–	–	−0.067^***^	−0.046^**^
	–	–	(0.00)	(0.00)
Job	−0.06^***^	−0.004	−0.026	−0.043^***^
	(0.00)	(0.00)	(0.00)	(0.00)
Alcohol	−0.050^***^	−0.010	−0.042^**^	−0.050^***^
	(0.00)	(0.01)	(0.01)	(0.00)
Smoke	0.025	0.017	0.019	0.030^*^
	(0.00)	(0.01)	(0.01)	(0.00)
Education	0.029^*^	0.024	0.057^***^	0.011
	(0.00)	(0.00)	(0.00)	(0.00)
Age	−0.141	−0.497^***^	−0.255^**^	−0.280^***^
	(0.00)	(0.00)	(0.00)	(0.00)
Age^∧^2	0.191^**^	0.571^***^	0.342^***^	0.322^***^
	(0.00)	(0.00)	(0.00)	(0.00)
Having adequate dietary knowledge literacy	−0.042^**^	−0.016	−0.047^**^	−0.016
	(0.01)	(0.00)	(0.00)	(0.00)
Income	−0.020	−0.011	−0.011	−0.027^*^
	(0.00)	(0.00)	(0.00)	(0.00)
Residence	−0.006	0.026	0.000	0.000
	(0.00)	(0.00)	(.)	(.)
Marriage	0.016	0.014	0.017	0.018
	(0.01)	(0.00)	(0.01)	(0.01)
Knowing about CFP/DGGCR	0.014	0.021	0.023	0.008
	(0.01)	(0.00)	(0.00)	(0.00)
Hypertension	0.172^***^	0.104^***^	0.146^***^	0.148^***^
	(0.01)	(0.00)	(0.01)	(0.00)
Constant	0.060^**^	0.084^***^	0.042	0.115^***^
	(0.03)	(0.02)	(0.03)	(0.02)
Observation	4,480	4,081	3,334	5,227

*Robust standard errors are reported in parentheses*.

* *10% significance level*.

** *5% significance level*.

*** *1% significance level*.

## Discussion

The present study was designed to determine the effect of the complex relationships among Chinese adults' dietary knowledge, socioeconomic factors, and stroke, and data from the large population survey of CHNS in 2015 were used. We used the OLS to estimate the relationships among dietary knowledge, SES, and stroke. We found that the risk of stroke decreases significantly with increasing dietary knowledge, and this result is consistent with the results of previous studies and reflects the results of Zhou et al. (20), Bonaccio et al. (42), and De Vriendt et al. (43), who found that improving residents' dietary knowledge can improve their eating habits and lifestyles. This approach is an important way to reduce the incidence of stroke and related chronic non-communicable diseases in the population throughout the life cycle. A rich and complete dietary knowledge system will effectively guide individuals to show scientific dietary behaviors, thereby helping them obtain a healthy body. Consequently, improving residents' knowledge of diet may help reduce the risk of stroke.

The risk of stroke in Chinese women is statistically significantly lower than that of men, as exemplified in the report of Xia et al. (44). After performing regression analysis on gender, we found that the impacts of dietary knowledge scores on different gender groups vary. Under the premise that other conditions remain unchanged, every time the female group's dietary knowledge score increases by one point, the risk of stroke will be significantly reduced by 7.7%. Najafi and Sheikhvatan (45) showed that the relationship between dietary patterns and risk of chronic diseases, such as hypertension, is inconsistent between genders. Women not only have richer dietary knowledge than men but are more willing to actively search information about food nutrition, whereas more than 25% of men show no interest (46). Influenced by traditional Chinese culture, women are mostly responsible for home cooking, and thus they pay more attention to reasonable diet to ensure the health of family members (47). Men often underperform in changing their own and families' dietary habits (48), and thus the relationship between the mastery of dietary knowledge and the risk of stroke is non-significant. Women pay more attention to their appearance and figures, and they have a strong focus on nutrition and health of food in their diet (49). Women play an important role in family life, allowing them to have health awareness and healthy behaviors, which will bring great benefits to the other members of their families. Hence, we should organize a variety of health education programs for women in a purposeful manner to effectively improve the lifestyles of other people.

China has experienced rapid economic growth over the past few decades, the social structure has undergone dramatic changes, and the level of urbanization has continued to expand. The rates of correct answers of rural residents with regard to dietary knowledge are significantly lower than those of urban residents. Notably, improvement in dietary knowledge can significantly reduce the prevalence of stroke among rural residents, but its impact on urban residents is non-significant. However, this study did not find a significant relationship between the dietary knowledge and health of urban residents. Nevertheless, good dietary preferences are significantly related to the reduction in the possibility of stroke in rural residents. A possible explanation for this result is that China is a developing country with a long-standing dual structure in urban and rural areas. The medical service level and coverage in urban areas are relatively high. Public medical care acts as a buffer against the health risks of urban residents (50). In sharp contrast, the income and food consumption levels of rural residents are significantly lower than that of urban residents, their nutritional status is slightly weaker and the risk of disease occurrence is higher (51). Given that medical services and coverage in rural areas are still inadequate, the health of rural residents is more affected by their SES, and education and income have considerable impacts on health. Owing to the development of the economy and the progress of the social level, the food processing industry has developed rapidly, the number of shops and vendors in rural areas have rapidly increased, and the dietary structures of rural residents have undergone significant changes (52). Currently, rural residents have insufficient intake of calories, carbohydrates, and proteins and excessive fat intake (52), which increase the risk of stroke and related diseases in rural residents. From the long-term goal of improving the overall nutritional status of rural residents, and efforts should be made to ensure dietary balance and improve nutritional awareness. Nutrition and health knowledge should be introduced to rural residents to enable them to optimize their dietary structures and to reduce their risk of suffering from chronic diseases, such as stroke.

After reform and opening up, Chinese residents have considerably increased incomes, and their consumption levels and quality of life have increased obviously. Different income groups have different levels of understanding of dietary knowledge, and high-income groups are more likely to have high levels of dietary knowledge (53). The current study found that the impact of dietary knowledge on stroke patients varies with income level. Low-income groups are more likely to suffer from stroke, but the more knowledgeable low-income groups are about diet, the more effectively they reduce their risk of stroke. A possible explanation for this finding is that the living habits of residents with different incomes are related to marginal utility. The effect of the law of diminishing marginal utility exists widely around us (54), and it affects daily life and study. According to this law, people with insufficient dietary knowledge can increase their reserves of relevant knowledge to improve their health. Lifestyle is likely an intermediate mechanism (55), and the impact of lifestyle on individual health is self-evident. Groups in different socioeconomic status may be due to differences in behavior and lifestyle, leading to differences in health status. High-income groups have higher levels of dietary knowledge and better medical resources, and a high income enables an individual to consume foodstuffs with high nutritional values, such as dairy products, seeds, and nuts. Thus, they have a scientific food consumption structures and balanced nutritional intake (53), which to a certain extent reduces the prevalence of stroke. People with high incomes may also have better eating habits, and improvement in dietary knowledge under the effect of diminishing marginal utility is not a relevant factor for promoting health. By contrast, low-income groups are more likely to get sick (56), and thus receiving correct dietary knowledge may improve their original dietary structures, particularly knowledge of ways to increase their intake of high-quality fats and proteins (57). This finding shows that the increase in dietary knowledge improves nutritional quality. This information can be used in developing interventions aimed at low-income residents. Increasing dietary knowledge is helpful in improving the nutritional status of a population and has a positive effect on the optimization of diet quality, likely improving health status and reducing the prevalence of non-communicable diseases, such as stroke.

Individuals with different educational levels present different levels of performance. Dietary knowledge has a significant impact on the reduction of stroke incidence in people with low educational levels but has little effect on the reduction of the risk of diseases in people with high educational levels. Education has a significant positive impact on health when demographic factors, such as gender and age, are controlled (58), and thus people with high levels of education are more likely to have healthy bodies and longer life expectancy (59). Meara et al. (60) found that the gap in the health of people with different educational levels has widened in the past few decades. In addition, education has a cumulative effect on health, that is, people with high educational levels are not only healthier than those with lower educational levels but also the gap increases with age (59). People with high levels of education usually have better careers and higher incomes and can provide more favorable conditions for their own health. By contrast, people with high levels of education know more about health, and they are more likely to avoid unhealthy behaviors. Education significantly affects the SES of individuals (61), and groups with lower SES are more prone to psychological pressure, which in turn leads to poor health (62). Given that people with lower SES are more likely to face chronic and acute stressors, which greatly increase risk of chronic diseases, such as stroke (63). Therefore, the government should improve dietary knowledge and health education of populations with low levels of education.

This study had several limitations. First, although the CHNS is a large-scale demographic survey for China, its comprehensiveness is still inadequate to a certain extent. Second, our research uses cross-sectional data rather than panel data, and the occurrence of chronic diseases, such as stroke, is the result of the long-term effects of related risk factors. Cross-sectional data cannot well reflect the influence of SES and dietary knowledge on stroke. Therefore, in future investigations, panel data needed to conduct a prospective cohort study to determine how dietary knowledge affects the prevalence of stroke in Chinese adults. Furthermore, stroke is related to diseases, such as high blood pressure and hyperlipidemia (37). Being limited to survey data, this study lacks a detailed discussion on this relationship. Nevertheless, our study has strengths. This study explored the influence of dietary knowledge and SES on the prevalence of stroke by using multiple linear regression models and obtained some interesting results. These findings contribute in several ways to our understanding of the linear relationship between dietary knowledge, SES, and stroke in the Chinese population and make several contributions to the current literature.

## Conclusion

The aim of the present research was to use the CHNS data and multiple linear regression models to explore the complex relationships among stroke, dietary knowledge, and SES in Chinese adults. This study showed that improving dietary knowledge scores can effectively reduce the risk of stroke, and a certain degree of heterogeneity was found with regard to the impact of SES on residents suffering from stroke, particularly in gender, income, educational level, and urban-rural differences. Increase in dietary knowledge has a more obvious positive impact on female groups, low-income groups, low-education groups, and rural residents. Our future research will use panel data and include more potential related factors to discuss the causes of stroke further.

The findings of this study have a number of important implications for future practice. Therefore, the Chinese government must adopt corresponding healthy diet education strategies for people with different SES. The government should shift more work centers to disadvantaged groups, and policies should be focused on rural areas. This approach may improve the diet quality of the society as a whole and reduce the risk of stroke. The mechanism of dietary knowledge and SES in stroke must be investigated further.

## Data Availability Statement

Publicly available datasets were analyzed in this study. This data can be found here: cpc.unc.edu/projects/china.

## Author Contributions

RX: conceptualization, software, data curation, and supervision. XZ: methodology and writing—review and editing. QZ: formal analysis and visualization. ZW: investigation, project administration, and funding acquisition. RX and ZW: writing—original draft preparation. All authors have read and agreed to the published version of the manuscript.

## Funding

This research was funded by Science and Technology Base and Talent Project of Guangxi Province of China (Grant NO. GuikeAD20238071).

## Conflict of Interest

The authors declare that the research was conducted in the absence of any commercial or financial relationships that could be construed as a potential conflict of interest.

## Publisher's Note

All claims expressed in this article are solely those of the authors and do not necessarily represent those of their affiliated organizations, or those of the publisher, the editors and the reviewers. Any product that may be evaluated in this article, or claim that may be made by its manufacturer, is not guaranteed or endorsed by the publisher.

## Footnotes

^1^CHNS's original data, related survey content and quality control details can be found on its official website (cpc.unc.edu/projects/china).
