# Supplementary material for: Relationship Between Dietary Knowledge, Socioeconomic Status, and Stroke Among Adults Involved in the 2015 China Health and Nutrition Survey
Source: Front Nutr. 2021 Sep 27;8:728641. doi: 10.3389/fnut.2021.728641 (PMC8502799; doi:10.3389/fnut.2021.728641)
Supplement: Supplementary file 1 [file Data_Sheet_1.pdf]

# *Supplementary Material*

**Table A1.** Diet knowledge questionnaire

| NO. | Do you strongly agree, somewhat agree, somewhat disagree or strongly disagree with this statement? | True/False |
|---|---|---|
| Q1 | Choosing a diet with a lot of fresh fruits and vegetables is good for one's health. | T |
| Q2 | Eating a lot of sugar is good for one's health. | F |
| Q3 | Eating a variety of foods is good for one's health | T |
| Q4 | Choosing a diet high in fat is good for one's health. | F |
| Q5 | Choosing a diet with a lot of staple foods rice and rice products and wheat and wheat products is not good for one's health. | T |
| Q6 | Consuming a lot of animal products daily (fish, poultry, eggs and lean meat) is good for one's health. | F |
| Q7 | Reducing the amount of fatty meat and animal fat in the diet is good for one's health. | T |
| Q8 | Consuming milk and dairy products are good for one's health. | T |
| Q9 | Consuming beans and bean products are good for one's health. | T |
| Q10 | Physical activities are good for one's health. | T |
| Q11 | Sweaty sports or other intense physical activities are not good for one's health. | F |
| Q12 | The heavier one's body is, the healthier he or she is. | F |
| Q13 | Eating salty foods can cause hypertension. | T |
| Q14 | Refined grains (rice and wheat flour) contain more vitamins and materials than unrefined grains. | F |
| Q15 | Lard is healthier than vegetable oils. | F |
| Q16 | Vegetables contain more starch than staple foods (rice or wheat flour). | F |
| Q17 | Eggs and milk are the important sources of high-quality protein. | T |

**Table A2.** Food preferences questionnaire

| NO. | How much do you like this food: Like very much, like somewhat, dislike somewhat, or dislike very much? | Positive/Negative |
|---|---|---|
| Q1 | Fast food (KFC, pizza, hamburgers, etc.) | N |
| Q2 | Salty snack foods (potato chips, pretzels, French fries, etc.) | N |
| Q3 | Fruits | P |
| Q4 | Vegetables | P |
| Q5 | Soft drinks and sugared fruit drinks | N |

**Table A3.** Explanation of each variable and detailed processing process

| Variable | Unit | Definition |
|---|---|---|
| Stroke | —— | 1 = stroke, 0 = health |
| Diet knowledge | —— | Accumulation of each answer |
| Food preference | —— | Accumulation of each answer |
| Gender | —— | 1 = female, 0 = male |
| Job | —— | 1 = in work, 0 = jobless |
| Alcohol | —— | 1 = drinker, 0 = no alcohol |
| Smoke | —— | 1 = smoker, 0 = non-smoker |
| Education | —— | 1 = graduated from primary school,2 = lower middle school degree,3 = technical or vocational degree or upper middle school degree,4 = university or college degree or higher |
| Age | year | Age |
| Age^2 | $year^2$ | Age squared |
| Having adequate dietary knowledge literacy | —— | 1 = yes, 0 = no |
| Income | CNY | Annual household income. Among them, 53 individual households have negative annual incomes. The reason may be that these households have debts. |
| Residence | —— | 1 = city, 0 = countryside |
| Marriage | —— | 1 = married, 0 = otherwise |
| Knowing about CFP/DGGCR | —— | 1 = yes, 0 = no |
| Hypertension | —— | 1 = hypertension, 0 = health |
